# Investigating temporal and prosodic markers in clinical high‐risk for psychosis participants using automated acoustic analysis

**DOI:** 10.1111/eip.13357

**Published:** 2022-10-07

**Authors:** Bianca Bianciardi, Ruchika Gajwani, Joachim Gross, Andrew I. Gumley, Stephen M. Lawrie, Melina Moelling, Matthias Schwannauer, Frauke Schultze‐Lutter, Alessio Fracasso, Peter J. Uhlhaas

**Affiliations:** ^1^ Institute of Neuroscience and Psychology University of Glasgow Glasgow UK; ^2^ School of Health and Wellbeing University of Glasgow Glasgow UK; ^3^ Institute for Biomagnetism and Biosignalanalysis University of Muenster Muenster Germany; ^4^ Department of Psychiatry Univ. of Edinburgh Edinburgh UK; ^5^ Department of Clinical Psychology Univ. of Edinburgh Edinburgh UK; ^6^ Department of Psychiatry and Psychotherapy, Medical Faculty Heinrich Heine University Düsseldorf Germany; ^7^ Department of Psychology, Faculty of Psychology Airlangga University Surabaya Indonesia; ^8^ University Hospital of Child and Adolescent Psychiatry and Psychotherapy University of Bern Bern Switzerland; ^9^ Department of Child and Adolescent Psychiatry Charité Universitätsmedizin Berlin Germany

**Keywords:** automated acoustic analysis, clinical high‐risk, early psychosis, prosody, speech

## Abstract

**Aim:**

Language disturbances are a candidate biomarker for the early detection of psychosis. Temporal and prosodic abnormalities have been observed in schizophrenia patients, while there is conflicting evidence whether such deficits are present in participants meeting clinical high‐risk for psychosis (CHR‐P) criteria.

**Methods:**

Clinical interviews from CHR‐P participants (*n* = 50) were examined for temporal and prosodic metrics and compared against a group of healthy controls (*n* = 17) and participants with affective disorders and substance abuse (*n* = 23).

**Results:**

There were no deficits in acoustic variables in the CHR‐P group, while participants with affective disorders/substance abuse were characterized by slower speech rate, longer pauses and higher unvoiced frames percentage.

**Conclusion:**

Our finding suggests that temporal and prosodic aspects of speech are not impaired in early‐stage psychosis. Further studies are required to clarify whether such abnormalities are present in sub‐groups of CHR‐P participants with elevated psychosis‐risk.

## 1 INTRODUCTION

Language abnormalities are widely established in schizophrenia (Andreasen & Grove, 1986). More recently, acoustic speech parameters, such as prosodic and temporal variables, have been investigated and found to be impaired in Schizophrenia (Cohen et al., 2014; Parola et al., 2020). Prosodic features include pitch variation, vowel space and intensity (Zhang, 2016), while temporal variables correspond to the absence/presence of speech signal, such as pause length and speech duration, and the number of such events, that is, pauses, syllables, articulation rate (Wennerstrom, 2001).

To date, few studies examined acoustic impairments in participants meeting clinical high‐risk for psychosis (CHR‐P) criteria. Temporal impairments included increased number and duration of pauses which were associated with negative symptoms (Stanislawski et al., 2021), while aberrant pauses in turn‐taking correlated with positive symptoms in CHR‐Ps (Sichlinger et al., 2019).”Acoustic abnormalities were pronounced in CHR‐Ps who transitioned to psychosis (Agurto et al., 2020). These findings suggest that acoustic metrics may constitute biomarkers for early detection and diagnosis (Corcoran et al., 2020). However, it is currently unclear whether acoustic impairments are characteristic of the CHR‐P status in general or whether temporal or prosodic abnormalities are only present in CHR‐Ps with elevated psychosis‐risk (Agurto et al., 2020).

The current study aimed to address this question by assessing prosodic and temporal variables in CHR‐Ps. Acoustic metrics were compared to participants with substance use and affective disorders (clinical high‐risk negative; (CHR‐N) and to healthy controls (HCs)). Additionally, we addressed methodological issues, such as the duration of speech samples, on acoustic estimates.

## 2 METHODS

### 2.1 Participants

CHR‐Ps were recruited as part of the “Youth Mental Health Risk and Resilience (YouR) Study” (Uhlhaas et al., 2017). CHR‐P criteria were assessed through the positive items of the Comprehensive Assessment of At‐Risk Mental States (CAARMS‐p; Yung et al., 2005) and the Schizophrenia Proneness Instrument, Adult version (SPI‐A; Schultze‐Lutter et al., 2007) and included: Attenuated Psychosis Symptoms, Brief Limited Intermittent Psychotic Symptoms, Genetic Risk and Deterioration Syndrome and COGDIS/COPER criteria. CHR‐Ps were excluded if they had a history of psychotic disorders. Our CHR‐P sample was not taking antipsychotic medication.

### 2.2 Speech acquisition

Recordings were obtained from baseline CAARMS‐p interviews (Yung et al., 2005) using a single cardioid microphone. Our sample consisted of 50 CHR‐Ps, 23 CHR‐Ns and 17 HCs. Audios were manually separated into two different files containing the participant's and interviewer's speech using Audacity® (Audacity Team, 2021). Pauses resulting from a switch between speakers were assigned to the individual that started speaking after the pause. Speech segments where both speakers spoke simultaneously were removed. Participants' audios with durations below 2 minutes were excluded for further analysis. Recordings were denoised using the Accusonus ERA Bundle 5.0 plug‐in and normalized to set the audio's amplitude to an absolute peak of 0.99 (Praat Vocal toolkit: Normalize script; Corretge, 2012). The features extracted were grouped into temporal (de Boer et al, 2020) and prosodic (Agurto et al., 2020) measures.

### 2.3 Temporal features

Speech and articulation rates were obtained from the “Praat Script Syllable Nuclei v2” (Quené et al., 2011). Raw measures were adjusted to the duration of the participant audio track or to the total interview duration (only where specified). Temporal features extracted: articulation rate, speech rate, pause rate, average syllable and pause duration, mean length of runs, percentage of time pausing and articulating, percentage of time pausing and articulating (relative to the total interview duration, hereafter: adjusted)(Supporting Table 1).

### 2.4 Prosodic features

Only the participant's speech was utilized for prosodic analysis. Using the Praat Vocal Toolkit (Corretge, 2012) we extracted: glottal pulse period, jitter, shimmer, harmonics‐to‐noise ratio (HNR), noise‐to‐harmonics ratio (NHR), voice breaks and unvoiced frames. Additional pitch analysis made use of a previously implemented Praat script for pitch extraction (Lennes et al., 2016). All pitch values were converted from Hz to semitones (ST; relative to 100 Hz frequency; Supporting Table 2).

### 2.5 Statistical analysis

All statistical analyses were performed using R (version 4.0.5) with statistical significance set at p < .05 (Lakens et al., 2018). Group differences were analysed using one‐way ANOVAs for continuous variables and non‐parametric Kruskal‐Wallis H tests for ordinal variables followed by post‐hoc tests. Non‐parametric Kendall's Tau Coefficient correlations assessed the association between the acoustic variables and symptom severity/functioning in CHR‐Ps. *Antidepressant medications (ADMs)* can impair phono‐articulation (Stassen et al., 1998) and could represent a confounding factor in our analysis. Post‐hoc linear regressions were performed including group status (Diagnosis: CHR‐P vs CHR‐N), medication *(ADMs or no*‐*ADMs)* and their interaction as predictors on acoustics. Due to significant group differences in the interview duration (CHR‐P, 31 min; HCs, 15 min; and CHR‐N, 26 min), we used linear regression to remove the influence of interview duration from each dependent variable and examined the residual of the model as the corrected dependent variable, resulting in corrected and uncorrected speech data.

## 3 RESULTS

CHR‐Ps presented increased symptom severity and distress in their total positive CAARMS‐p scores and all subscales compared to HCs and CHR‐Ns as well as in SPI‐A total score (all *p* < .001, uncorrected). CHR‐Ps showed lower functioning scores than CHR‐Ns and HCs (*p* < .001) (Supporting Table 3).

Uncorrected results showed significantly higher speech rate (*p* = .012) and lower mean length of runs (*p* = .009) for CHR‐Ps compared to CHR‐Ns (Supporting Table 4). Group differences were observed for average pause duration (*p* = .03), percentage of time articulating (p = .041) and pausing (*p* = .041), which did not survive multiple comparison corrections. Prosody data revealed higher unvoiced frame percentage for CHR‐Ns compared to CHR‐Ps (p = .003). The corrected data showed that CHR‐Ps had significant higher speech rates (*p* = .019) and lower mean length of runs (*p* = .022) compared to CHR‐Ns (Supporting Table 5). Additionally, CHR‐Ps had a significantly lower percentage of total time pausing compared to CHR‐Ns (*p* = .048). Prosodic measures yielded significant difference across groups only for unvoiced frame percentage (*p* = .001), which was higher for CHR‐Ns compared to CHR‐Ps and HCs. Group status (CHR‐Ps vs. CHR‐Ns) remained a significant predictor of the acoustic variables after controlling for *ADM‐status* (Supporting Table 6). Significant correlations were obtained between the total CAARMS‐p severity and the following prosodic indices: 25th percentile pitch (*τ* = −0.27, *p* = .007) and 5th percentile pitch (*τ* = −0.29, *p* = .003). A significant association was also observed between total SPI‐A severity and 25th percentile pitch (*τ* = −0.26, *p* = .009). However, no correlation remained significant following FDR correction.

## 4 DISCUSSION

We investigated temporal and prosodic speech features to identify whether acoustic parameters are impaired in CHR‐Ps. Our data show that the CHR‐P group presented faster speech rate, more efficient speech production and less time spent pausing (temporal features) and lower unvoiced frames (prosodic index) compared to CHR‐Ns, while there were no differences compared to HCs, suggesting that acoustic speech parameters are intact in CHR‐Ps.

These findings contrast with previous studies that reported acoustic deficits in CHR‐P group (Agurto et al., 2020; Sichlinger et al., 2019). Impaired acoustic signatures were only found in CHR‐Ps with elevated positive symptoms and in those who transitioned to psychosis (Agurto et al., 2020; Sichlinger et al., 2019). However, similarly to the present results, no group effect was observed between the entire CHR‐P cohort and HCs (Sichlinger et al., 2019). We also did not observe robust associations between acoustic and clinical/functional variables. A previous study found that acoustic features were associated with negative symptoms in CHR‐P participants (Stanislawski et al., 2021). Our study and Sichlinger et al. (2019) elicited speech from clinical interviews, whilst Agurto et al. (2020) and Stanislawski et al. (2021) employed open‐ended interviews. The different acquisition protocols might explain the divergent findings, given that the context and content of interview settings significantly influence speech parameters (Cohen et al., 2016).

We observed impaired acoustic performance in CHR‐N compared to CHR‐Ps. This contrasts with previous findings that observed similar patterns of acoustic impairments across individuals with psychosis or other psychopathologies (Cannizzaro et al., 2004).

In schizophrenia, acoustic indices are generally associated with the presence of negative symptoms (Cohen et al., 2020). However, negative symptoms were not assessed in the current study. Importantly, acoustic findings in the current study were invariant to interview length, in line with previous results (Alghowinem et al., 2013). However, clinical interviews, while widely used in the literature (Sichlinger et al., 2019; Tahir et al., 2019), have several limitations, for example, different arousal levels that could impact speech parameters. Future studies should consider employing more controlled speech acquisition paradigms (Cohen et al., 2020).

In addition, CHR‐P participants in the YouR‐study were largely recruited from the community (McDonald et al., 2019). Clinical and community‐recruited CHR‐Ps may differ in transition rates (Fusar‐Poli et al., 2016) and thus our sample may be less‐psychosis enriched. However, CHR‐Ps in the YouR‐cohort were characterized by cognitive, clinical and physiological alterations that are consistent with the previous findings from CHR‐P cohorts recruited through clinical pathways (Grent et al., 2021; Haining et al., 2020, 2021).

In summary, our finding suggests that temporal and prosodic features are not impaired in CHR‐Ps. Given more robust evidence for semantic/syntactic impairments (Elvevåg et al., 2007; Gupta et al., 2018), these features may constitute more promising biomarkers for early detection and diagnosis. Future studies should clarify whether acoustic abnormalities are present in subgroups of CHR‐Ps with elevated psychosis‐risk.

## Supporting information


**Supporting Table 1** Temporal features

**Supporting Table 2** Prosodic features

**Supporting Table 3** Demographic and clinical/functional characteristics

**Supporting Table 4** Group comparison for acoustic variables uncorrected by interview duration

**Supporting Table 5** Group comparison for acoustic variables corrected by interview duration

**Supporting Table 6** Linear regression on the influence of *ADMs* on speech parameters between CHR‐Ps and CHR‐Ns

## Data Availability

Data sharing is not applicable to this article as no new data were created or analyzed in this study.
