# Supplementary material for: Investigating temporal and prosodic markers in clinical high‐risk for psychosis participants using automated acoustic analysis
Source: Early Interv Psychiatry. 2022 Oct 7;17(3):327–30. doi: 10.1111/eip.13357 (PMC10946925; doi:10.1111/eip.13357)
Supplement: Supplementary file 1 — Supporting Table 1 Temporal features [file EIP-17-327-s002.pdf]

Supporting Table 1

Temporal features

Variable	Definition/calculation	Measures
**Articulation rate**	Syllables / phonation time	*Speed fluency*. Speed in speech production.
**Speech rate**	Syllables / speech time (including pauses)	*Speed fluency*. Syllables spoken per seconds
**Average syllable duration**	Phonation time / syllables	*Speed fluency*. Average duration of syllables
**Mean length of runs**	Number of silent pauses / Number of syllables	*Speed fluency*. Efficiency in speech production
**Pause rate**	Total number of pauses / speaking time	*Breakdown fluency.* Number of pauses per minute
**Average pause duration**	Pause time / number of pauses	*Breakdown fluency.* Mean length of pauses
**Percentage of time articulating**	(Phonation time / participant interview duration) *100	*Breakdown fluency.* Duration of participant's speech
**Percentage of time pausing**	(Pause time / participant interview time) *100	*Breakdown fluency.* Duration of participant's pauses
**Percentage of time articulating (adjusted)**	(Phonation time / total interview duration) *100	*Breakdown fluency.* Duration of participant speaking based on the total interview
**Percentage of time pausing (adjusted)**	(Pause time/total interview time)*100	*Breakdown fluency.* Duration of participant's pauses based on the total interview
**Percentage of total time speaking**	(Speech time/total interview time)*100	*Breakdown fluency.* Reflects spontaneity or willingness to speak
**Participant/Interviewer ratio**	Participant phonation time /interviewer phonation time *100	*Breakdown fluency.* Reflects speech fluency or willingness to speak

Legend: Adjusted, relative to the total interview duration.
