# Supplementary material for: Investigating temporal and prosodic markers in clinical high‐risk for psychosis participants using automated acoustic analysis
Source: Early Interv Psychiatry. 2022 Oct 7;17(3):327–30. doi: 10.1111/eip.13357 (PMC10946925; doi:10.1111/eip.13357)
Supplement: Supplementary file 2 — Supporting Table 2 Prosodic features [file EIP-17-327-s003.pdf]

Supporting Table 2

Prosodic features

**Pitch variation**		
**Variable**	**Description/calculation**	**Statistical descriptors**
**Pitch**	Repetitive opening-closing sequence of vocal cords. Autocorrelation-based pitch algorithm. Pitch in semitones (relative to 100 Hz).	Median, skewness, kurtosis, 5_{th}, 25_{th}, 75_{th}, 95th, percentiles and Interquartile range(IQR).
**Glottal pulse period**	Variance in voice quality affected by folds of the vocal cords when speaking	Mean, SD

**Voice quality**		
**Variable**	**Description/calculation**	**Statistical descriptors**
**Jitter**	Fluctuations in pitch	Local absolute, ppq5
**shimmer**	Fluctuations in volume	Local dB value, apq5
**Voice Breaks**	Measures the maintenance in phonation during speech	Percentage of locally unvoiced frames, percentage of voice breaks
**Harmonics to Noise Ratio**	Efficiency in speech. Ratio vibration of vocal cords/glottal noise (dB)	HNR, NHR

Legend: SD, standard deviation; apq5, five-point Amplitude Perturbation Quotient; ppq5, five-point Period Perturbation Quotient; dB, decibels; HNR, Harmonics to noise ratio; NHR, Noise to harmonics ratio.
