# Supplementary material for: Investigating temporal and prosodic markers in clinical high‐risk for psychosis participants using automated acoustic analysis
Source: Early Interv Psychiatry. 2022 Oct 7;17(3):327–30. doi: 10.1111/eip.13357 (PMC10946925; doi:10.1111/eip.13357)
Supplement: Supplementary file 3 — Supporting Table 3 Demographic and clinical/functional characteristics [file EIP-17-327-s001.pdf]

Supporting Table 3

Baseline characteristics of CHR-Ps, CHR-Ns and HCs

Characteristic	CHR-Ps (n = 50)	HCs (n =17)	CHR-Ns (n = 23)	df	F/ χ2/H	p	Post Hoc Contrasts †
**Age (years), M ± SD**	21.1 (3.92)	22.4 (3.74)	22.5 (4.88)	2	F = 1.21	.303	
**Gender, N female (%)**	39 (78.0)	11 (64.7)	17 (73.9)	2	χ2 = 1.18	.554	
**Years of education, M ± SD**	14.9 (2.74)	16.8 (3.72)	16.8 (4.26)	2	F = 3.365	.039*	
**UK Citizen, N (%)**	34 (68)	10 (58.8)	13 (57)	2	χ2 = 1.08	0.58	
**GAF, median (range)**	61.5 (42-95)	89 (68-96)	78 (43-94)	2	H = 26.3	< .001 ***	1&2,1&3
**CAARMS Positive Items, median (range)**							
Unusual Thought Content	0 (0-5)	0 (0)	0 (0-3)	2	H = 12.351	< .001 **	1&2
Non-Bizarre Ideas	3.5 (0-6)	0 (0-2)	0 (0-2)	2	H = 25.502	< .001 ***	1&2,1&3

Perceptual Abnormalities	3 (0-5)	0 (0-3)	0 (0-3)	2	H = 29.808	< .001 ***	1&2,1&3
Disorganised Speech	1 (0-4)	0 (0)	0 (0-2)	2	H = 16.947	< .001 ***	1&2,1&3
**Total Positive Severity**	23.5 (0-56)	0 (0-12)	4 (0-13)	2	H = 44.597	< .001 ***	1&2,1&3
**CHR Criteria Subgroup, N (%)**							
**UHR**	18 (36)	0 (0)	0 (0)				
**BS**	18 (36)	0 (0)	0 (0)				
**UHR/BS**	13 (26)	0 (0)	0 (0)				
**GF: Social, median (range)**	8 (5-9)	9 (8-9)	8 (7-9)	2	H = 14.529	< .001 ***	1&2
**GF: Role, median (range)**	8 (4-9)	9 (7-9)	8 (7-9)	2	H = 10.762	.005 ***	1&2
**SPIA severity, median (range)**	6 (0-37)	0 (0-2)	0 (0-10)	2	H = 38.617	<. 001 ***	1&2,1&3
**Medication, N (%)**				6	$\chi 2$ = 16.849	.009 *	
Anti-depressants	8 (16)	0 (0)	16 (69.6)				
Other	5 (10)	0 (0)	0 (0)				
Multiple	6 (12)	0 (0)	0 (0)				

**Clinical characteristics, N (%)**			
Anxiety disorders	34 (68)	0 (0)	13 (57)
Mood disorders	30 (60)	0 (0)	5 (21.7)
Eating disorders	4 (0.8)	0 (0)	1 0.4)
Suicide Risk	24 (48)	1 (0.6)	4 (17.4)
Alcohol Dependence/Abuse	12 (24)	0 (0)	5 (21.7)
Substance Dependence/Abuse	4 (0.8)	0 (0)	1 (0.4)

† CHR-P = 1, HC = 2, CHR-N = 3. P-values: * $p < .05$; ** $p < .01$; *** $p < .001$

Legend: CHR-P, clinical high-risk for psychosis; CHR-N, clinical high-risk-negative; HC, healthy control; H, Kruskal-Wallis H test; F, F value (ANOVA); $\chi 2$, chi-square test; p, p-value; df, degrees of freedom; N/n, sample size; M, mean; SD, standard deviation; GAF, Global Assessment of Functioning; UHR, ultra-high risk; BS, basic symptoms; GF, Global Functioning; SPI-A, ; CAARMS, Comprehensive Assessment of At-Risk Mental States.
