# Supplementary material for: Investigating temporal and prosodic markers in clinical high‐risk for psychosis participants using automated acoustic analysis
Source: Early Interv Psychiatry. 2022 Oct 7;17(3):327–30. doi: 10.1111/eip.13357 (PMC10946925; doi:10.1111/eip.13357)
Supplement: Supplementary file 4 — Supporting Table 4 Group comparison for acoustic variables uncorrected by interview duration [file EIP-17-327-s004.pdf]

Supporting Table 4

Group comparison for acoustic variables uncorrected by interview duration

Variable	CHR (n = 50)		HCs (n = 17)		CHR-N (n = 23)				Post Hoc Contrasts †
	Median	Range	Median	Range	Median	Range	H	p	
**TEMPORAL VARIABLES**									
**Speech rate**	2.54	(0.90-3.89)	2.28	(1.10-3.35)	1.95	(0.82 - 3.54)	8.86	.012*	1&3
**Articulation rate**	4.28	(3.23 - 5.19)	4.29	(3.68 - 4.91)	3.96	(1.92 - 4.71)	5.03	.081	
**Average syllable duration**	0.23	(0.19 - 0.31)	0.23	(0.20- 0.27)	0.25	(0.21- 0.52)	5.15	0.07	
**Average pause duration**	1.027	(0.76-1.47)	1.08	(0.91- 1.87)	1.09	(0.84-2.08)	6.88	0.03*	/
**Mean length of runs**	0.15	(.056-0.56)	0.201	(0.07- 7 0.37)	0.23	(0.075-0.43)	9.34	0.009**	1&3
**Pause Rate**	0.64	(0.22-1.82)	0.81	(0.35 - 1.82)	0.77	(0.34-1.56)	5.31	.070	
**Percentage of time articulating**	61.16	(27.79-83.92)	53.06	(22.66-74.18)	56.99	(25.93-76.91)	6.40	.041*	/
**Percentage of time pausing**	38.84	(16.08 - 72.20)	46.94	(25.81-77.33)	43.01	(23.09-74.06)	6.40	.041*	/
**Percentage of time articulating (adjusted)**	26.72	(5.84 - 70.18)	19.58	(3.37 - 37.84)	25.61	(10.18 - 53.69)	5.66	0.059	

**Percentage of time pausing (adjusted)**	16.29	(7.07-39.89)	16.73	(7.07-39.89)	18.43	(7.63 - 58.59)	4.01	0.134	
**Percentage of total time speaking**	47.004	(12.91-91.79)	37.91	(14.87-68.44)	44.87	(17.80-96.09)	4.59	0.10	
**PROSODIC VARIABLES**									
**Mean Pulses**	0.005	(0.004-0.009)	0.005	(0.003-0.009)	0.005	(0.004-0.009)	2.61	.272	
**SD Pulses**	0.001	(0.0005-0.004)	0.001	(0.0005-0.003)	0.001	(0.0004-0.009)	2.10	0.34	
**Jitter**	0.0001	(0.0005 - 0.0003)	0.0001	(0.00006-0.0003)	0.0001	(0.0007-0.0003)	0.84	.655	
**Jitter ppq5**	1.24	(0.6-2.83)	1.37	(0.9-3.09)	1.15	(0.77-2.07)	0.84	0.647	
**Shimmer local dB**	1.29	(0.89-1.79)	1.28	(0.99-1.78)	1.25	(0.99-1.47)	0.433	0.805	
**Shimmer apq5**	8.30	(4.72-13.89)	8.55	(5.88-13.82)	8.56	(6.02-10.64)	0.096	0.953	
**Voice breaks**	51.82	(34.52-74.45)	54.90	(43.47-65.37)	58.12	(40.09-82.3)	5.25	0.07	
**Unvoiced frames (%)**	45.67	(29.76 - 68.62)	49.47	(40.04-58.33)	52.19	(35.45-79.84)	7.29	.003**	1&3
**NHR**	0.19	(0.1-8 0.42)	0.21	(0.13- 0.43)	0.19	(0.11-0.36)	1.797	0.407	
**HNR**	10.89	(5.17-15.8)	10.16	(5.1- 14.0)	10.36	(6.29-14.72)	0.813	0.666	
**Pitch Median ST**	11.81	(7.38-25.59)	11.98	(7.63-22.85)	11.43	(5.88-14.47)	2.56	.278	
**Pitch Skewness**	1.68	(-0.51-3.96)	1.56	(-0.61-4.08)	2.14	(0.24-3.28)	3.53	.171	

**Pitch Kurtosis**	7.39	(1.27-21.53)	5.36	(1.36-24.13)	9.23	(1.45-30.07)	3.14	.208
**Pitch 5th pct**	-2.13	(-23.82- -0.63	-2.65	(-23.06- -1.04)	-1.1	(-6.9- -0.5)	2.98	.225
**Pitch 25th pct**	-0.37	(-20.97-0.64)	-0.7	(-19.27-0.69)	-0.59	(-5.1-1.4)	2.08	.353
**Pitch 75th pct**	2.72	(-0.62-20.07)	2.6	(-0.33-22.41)	2.43	(0.58-20.39)	1.03	.596
**Pitch 95th pct**	9.49	(1.74-23.55)	14.0	(2.24-24.69)	10.83	(2.84-24.00)	0.18	.913
**Pitch IQR**	3.39	(1.54-20.35)	3.55	(2.16-21.71)	3.22	(1.21-19.09)	1.72	.424

† CHR-P = 1, HC =2, CHR-N = 3. P-values: * $p < .05$; ** $p < .01$; *** $p < .001$

Legend: CHR-P, clinical high-risk for psychosis; CHR-N, clinical high-risk-negative; HC, healthy control; n, sample size; H, Kruskal-Wallis H test ; adjusted, relative to the total interview duration; IQR, Interquartile Range; pct, percentile; ST, semitones; NHR, noise to harmonics ratio; HNR, harmonics to noise ratio; apq5, five-point Amplitude Perturbation Quotient; ppq5, five-point Period Perturbation Quotient; SD, standard deviation; dB, decibel.
