# Supplementary material for: Investigating temporal and prosodic markers in clinical high‐risk for psychosis participants using automated acoustic analysis
Source: Early Interv Psychiatry. 2022 Oct 7;17(3):327–30. doi: 10.1111/eip.13357 (PMC10946925; doi:10.1111/eip.13357)
Supplement: Supplementary file 5 — Supporting Table 5 Group comparison for acoustic variables corrected by interview duration [file EIP-17-327-s006.pdf]

Supporting Table 5

Group comparisons for acoustic variables corrected by interview duration

Variable	CHR-P (n = 50)		HC (n = 17)		CHR-N (n = 23)				Post Hoc Contrasts †
	Median	Range	Median	Range	Median	Range	H	p	
**TEMPORAL VARIABLES**									
**Speech rate**	0.14	(1.57-1.50)	-0.03	(1.11-1.03)	-0.35	(-1.76-0.96)	7.87	0.019*	1&3
**Articulation rate**	0.21	(-0.92-1.023)	0.11	(-0.52-0.71)	-0.21	(-2.24-0.58)	4.86	0.088	
**Average syllable duration**	-0.018	(-0.053-0.062)	-0.012	(0.04-0.028)	0.007	(-0.036-0.27)	4.64	0.098	
**Average pause duration**	-0.068	(-0.36-0.34)	-0.049	(-0.21-0.73)	-0.013	(-0.26-1.00)	4.63	0.099	
**Mean length of runs**	-0.026	(-0.13-0.39)	-0.004	(-0.12-0.17)	0.029	(-0.10 -0.27)	7.65	0.022*	1&3
**Pause Rate**	-0.062	(-0.51-1.13)	0.011	(-0.43-0.97)	-0.05	(-0.39 -0.87)	1.69	0.43	
**Percentage of time articulating**	3.39	(-31.51-21.44)	-1.03	(29.82-18.83)	-0.47	(-36.51-14.62)	3.133	0.209	
**Percentage of time pausing**	-3.39	(-21.45-31.50)	1.03	(-18.84-29.81)	0.48	(-14.63-36.50)	3.133	0.209	
**Percentage of time articulating (adjusted)**	1.22	(-17.35-18.05)	-0.78	(-12.79-15.57)	-1.24	(-23.71-16.64)	0.056	0.97	

**Percentage of time pausing (adjusted)**	-2.27	(-13.08 - 20.02)	-1.86	(-7.43 - 17.32)	1.25	(-8.59 - 40.20)	6.07	0.048*	1&3
**PROSODIC VARIABLES**									
**Mean Pulses**	-0.0005	(-0.002- 0.003)	-0.0006	(-0.002- 0.004)	-0.0003	(-0.001- 0.003)	2.66	.263	
**SD Pulses**	-0.0002	(-0.0008- 0.003)	-0.0001	(-0.0009- 0.002)	-0.0003	(-0.0009- 0.001)	1.93	.382	
**Jitter local absolute**	-0.00001	(-0.00008- 0.0001)	-0.00001	(- 0.00007- 0.0002)	-0.00001	(-0.00006- 0.0001)	0.16	.923	
**Jitter ppq5**	-0.055	(-0.79- 1.49)	-0.053	(-0.53- 1.71)	-0.13	(-0.58- 0.64)	0.78	.676	
**Shimmer local dB**	0.004	(-0.42- 0.49)	-0.030	(-0.31- 0.478)	-0.047	(-0.29- 0.17)	0.48	.787	
**Shimmer apq5**	-0.084	(-3.87- 5.38)	-0.066	(-2.74- 5.27)	-0.030	(-2.36- 2.09)	0.12	.94	
**Voice breaks**	0.40	(-5.32-5.37)	-0.24	(5.38- 3.61)	-0.25	(-4.17- 4.24)	5.58	.062	
**Unvoiced frames**	-3.09	(-17.81- 19.5)	-3.10	(-12.29- 7.53)	3.004	(-16.154 30.81)	8.89	.001**	1&3, 2&3
**NHR**	-0.02	(-0.12- 0.20)	-0.006	(-0.09- 0.214)	-0.017	(-0.105- 0.14)	1.12	.570	
**HNR**	0.395	(-5.3- 5.37)	-0.248	(-5.38- 3.61)	-0.249	(-4.167- 4.24)	0.69	.709	
**Pitch Median ST**	-0.31	(-4.62- 13.32)	-0.33	(-5.003- 10.27)	-0.33	(-6.62- 2.15)	1.14	.566	
**Pitch Skewness**	0.012	(-2.16- 2.32)	0.0002	(-2.12- 2.59)	0.365	(-1.39- 1.55)	2.48	.288	
**Pitch Kurtosis**	-1.30	(-7.73- 13.42)	-2.36	(-6.7- 16.65)	0.43	(-6.61- 21.6)	2.2	.33	

**Pitch 5th pct**	1.517	(-19.91-2.90)	1.25	(-19.27-3.06)	1.74	(-3.117-3.66)	1.82	.402
**Pitch 25th pct**	1.143	(-19.03-2.21)	1.21	(-17.42-2.78)	1.21	(-3.26-3.33)	0.71	.700
**Pitch 75th pct**	-1.54	(-5.15-15.61)	-2.004	(-4.83-17.82)	-2.030	(-4.008-15.87)	1.91	.385
**Pitch 95th pct**	-1.41	(-9.95-12.20)	2.01	(-9.62-12.86)	-0.73	(-8.57-12.33)	0.07	.965
**Pitch IQR**	-2.42	(-4.84-13.87)	-2.91	(-4.79-15.04)	-2.91	(-4.86-12.65)	2.03	.361

† CHR-P = 1, HC =2, CHR-N = 3. P-values: * $p < .05$; ** $p < .01$; *** $p < .001$

Legend: CHR-P, clinical high-risk for psychosis; CHR-N, clinical high-risk-negative; HC, healthy control; n, sample size; H, Kruskal-Wallis H test; adjusted, relative to the total interview duration IQR, Interquartile Range; pct, percentile; ST, semitones; NHR, noise to harmonics ratio; HNR, harmonics to noise ratio; apq5, five-point Amplitude Perturbation Quotient; ppq5, five-point Period Perturbation Quotient; SD, standard deviation; dB, decibel.
