# Supplementary material for: Investigating temporal and prosodic markers in clinical high‐risk for psychosis participants using automated acoustic analysis
Source: Early Interv Psychiatry. 2022 Oct 7;17(3):327–30. doi: 10.1111/eip.13357 (PMC10946925; doi:10.1111/eip.13357)
Supplement: Supplementary file 6 — Supporting Table 6 Linear regression on the influence of ADMs on speech parameters between CHR‐Ps and CHR‐Ns [file EIP-17-327-s005.pdf]

Supporting Table 6

Linear regression on the influence of ADMs on speech parameters between CHR-Ps and CHR-Ns

**Acoustic variables**	**Predictors**	**β**	**S.E.**	**t**	***p***
**Speech rate**	intercept	0.20	0.12	1.74	0.087
	Diagnosis (CHR_N)	-0.56	0.20	-2.82	0.007 **
	medication	-0.12	0.19	-0.64	0.524
	Diagnosis*medication	0.22	0.35	0.63	0.532
**Mean length of runs**	intercept	-0.03	0.015	-1.93	0.058
	Diagnosis (CHR_N)	0.07	0.026	2.69	0.009 **
	medication	0.03	0.024	1.27	0.209
	Diagnosis*medication	-0.05	0.045	-1.07	0.287
**Pause time (%; adjusted)**	intercept	-3.557	1.52	-2.34	0.022 *
	Diagnosis (CHR_N)	6.88	2.61	2.64	0.010 **
	medication	4.52	2.47	1.83	0.072
	Diagnosis*medication	-2.48	4.57	-0.54	0.589
**Unvoiced frames (%)**	intercept	-2.18	1.66	-1.31	0.194
	Diagnosis (CHR_N)	8.31	2.85	2.91	0.005 **
	medication	2.60	2.70	0.96	0.340
	Diagnosis*medication	-6.86	4.99	-1.37	0.174

P-values: * $p < .05$; ** $p < .01$; *** $p < .001$

Legend: CHR-P, clinical high-risk for psychosis; CHR-N, clinical high-risk-negative; *ADM*, antidepressant medication; adjusted, relative to the total interview duration; %, percentage; β, beta coefficient; S.E, standard error; t, t statistics; p, p-value.
